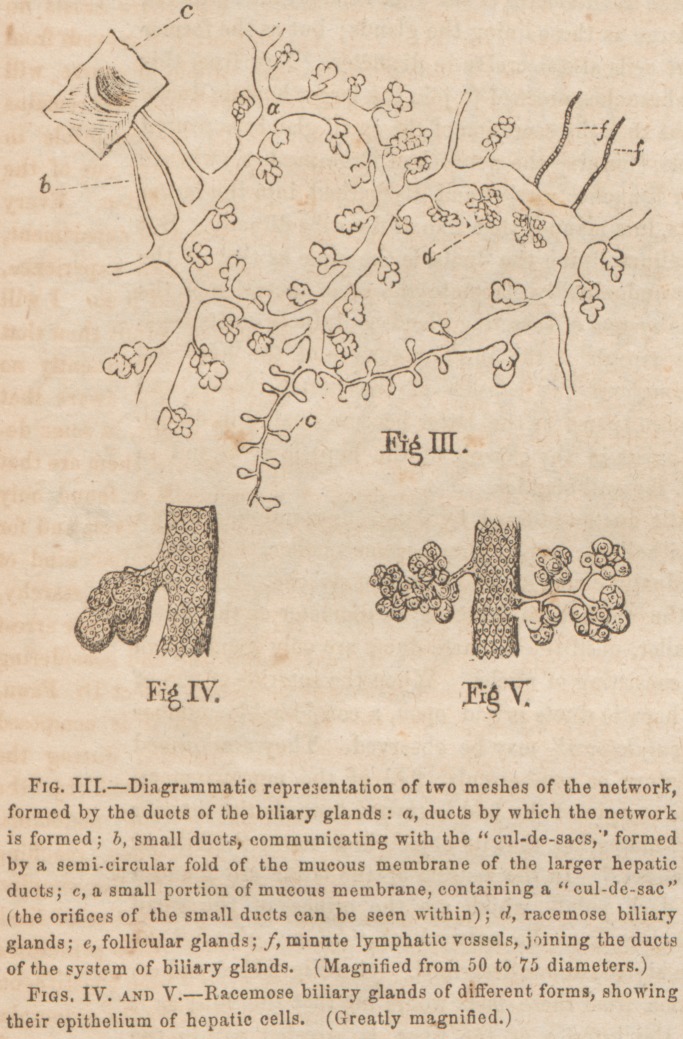# On the Microscopic Anatomy, Physiology and Pathology of the Human Liver

**Published:** 1864-04

**Authors:** H. D. Schmidt

**Affiliations:** Surgeon P. A. C. S.


### CONFEDERATE STATES MEDICAL & SURGICAL JOURNAL.

Vol. I. RICHMOND, APRIL, 1864. No. 4.

### ORIGINAL COMMUNICATIONS.

Art. I.—*On the Microscopic Anatomy, Physiology and Pathology of the Human Liver.* By H. D. Schmidt, Surgeon P. A. C. S. (Illustrated.)

[We commence the publication, in this number, of extracts from Dr. Schmidt's paper, confining ourselves more particularly to those passages where may be found the original views and observations of the author. The entire essay is worthy of perusal; and it is curtailed, simply because its length would exclude from our pages other matters of practical interest, which it is one of the principal objects of the Journal to circulate among its readers.—Ed.]

*On the Microscopic Anatomy of the Human Liver.*

The liver consists of a complex and intricate arrangement of arteries, veins, capillaries, hepatic cells, ducts, nerves, lymphatics, and also a system of small follicular and racemose glands, held together by a capsule of fibrous tissue. The blood by which it is supplied, is brought there by the portal vein and the hepatic artery, and carried off by the hepatic veins. The two former enter the organ at its inferior surface, after which they divide and sub-divide repeatedly, until their ultimate ramifications extend throughout its interior. The smallest branches of the latter originate in the parenchyma; by joining each other they form larger branches, which, in their turn, by often repeated junctions, form those few large trunks that join the vena cava at the posterior surface of the liver. The smaller branches of the hepatic artery and portal vein mostly run at right angles with those of the hepatic veins. The finest branches of the hepatic duct take their origin in the parenchyma in a manner that will be described hereafter. They also, by joining each other repeatedly, form larger ducts, until the common hepatic duct is formed. The latter issues from the organ at the same place where the hepatic artery and portal vein enter, and after having been joined by the duct of the gall-bladder, becomes the common bile duct, (ductus communis choledocus,) which empties into the duodenum. The portal vein, hepatic artery and hepatic duct are always found accompanying each other. The branches of the artery are often seen winding around those of the vein, and those of the duct still more frequently around those of the artery and vein. The duct, however, is usually near the artery, (see Fig. I. *a*, *b* and *d*.) In company with nerves, lymphatics and a system of small glands to be described hereafter, the portal vein, hepatic artery and hepatic duct are en-

ARMY MEDICAL LIBRARY WASHINGTON, D. C., U. S. A.

closed in a fibrous sheath. The latter has usually been known by the name of the "*capsule of Glisson.*" But in contradistinction to a similar one enclosing the hepatic veins, and to simplify the demonstration, I have termed it the "*capsule of the portal vessels.*" This sheath is analogous to the sheath of blood-vessels and nerves elsewhere, its office being only to hold the vessels, &c., together and impart strength to them. It is a continuation or process of the common fibrous capsule that surrounds the whole liver. A similar process proceeds from the latter to enclose the hepatic veins, which I have termed the "*capsule of the hepatic veins.*" We see, therefore, that the liver, like other organs, is surrounded by a fibrous capsule from which processes proceed into the interior to hold its component parts in their respective places; for it must be remembered that these capsular processes extend to enclose even the smallest branches of the vessels and duct.

If we examine the lungs, especially of some of the inferior animals, as the hog or sheep, we find that, independent of the pleura, they are surrounded by a fibrous capsule from which processes proceed, dividing the organ into smaller portions or lobules. In the latter animals, I have frequently separated these lobules without injuring, in the least, the parenchyma, as proved by subsequent minute injection. The separation is effected by carefully tearing the areolar tissue, (or processes of the common capsule,) by which the lobules are held together. This being done, the small portions remain only connected to each other by the bronchi and blood-vessels that enter. Thus, we see that the whole lung is actually composed of a number of small ones, the aggregate communicating by the ramifications of the bronchi and blood-vessels. Each individual lobule may be regarded as a lung in miniature. In man, and in most other animals, this separation is not so easily effected. Now in the liver of the hog and a few other animals this same arrangement exists; that is, from the capsule surrounding the whole organ, and those enclosing the blood-vessels and duct, small processes proceed to divide the parenchyma into small portions, similar to that in the lungs. These subdivisions have been termed by authors "*acini,*" and each of them represents a liver in miniature, just as in a racemose gland, each pouch is a gland in itself, though the ducts of many join to form one duct that belongs to all. The smaller branches of the blood-vessels and duct ramify between and surround the closed capsules of the acini until, at last, their finest branches penetrate the latter to form a communication with the parenchyma within.

On account of the above-mentioned arrangement in the liver of the hog, it has been regarded as more suitable for investigation, and thus has always been resorted to by investigators. And it is due to this fact that the idea of these subdivisions or "acini" still pervades the medical mind in connection with the human liver. I have already stated that acini exist in the liver of the hog and two or three other animals. In the human liver, as I shall show, no *acini* exist; *the capsules extend only over the ramifications of the vessels and duct, without entering the parenchyma for the purpose of subdivision;* and I repeat once more, that these capsules are analogous to the sheaths of blood-vessels, nerves, &c., elsewhere, with which they are also continuous externally to the organ.

The *parenchyma* of the liver consists of capillaries, hepatic cells, free nuclei and granules; the microscopic lymphatics, also, originate in it. Its anatomy is as follows: the finest branches of the portal vein and hepatic artery terminate in a net-work of capillary vessels; from the latter, the finest branches of the hepatic veins take their origin. Thus the blood of the portal vein and hepatic artery is carried to the same net-work of capillaries, where the arterial becomes mixed with the venous. After having traversed the capillaries, it is carried off by the hepatic veins and poured into the ascending vena cava. Besides this net-work of capillaries, through which the blood circulates, there exists another. They are independent of each other; that is, they do not communicate. In the latter, the finest branches of the hepatic duct and lymphatic vessels originate. The interspaces or meshes, formed by the two networks, are filled up by the hepatic cells, free nuclei, and granules. (See Figs. I. and II. and explanation.) The capillary vessels, forming the network that gives origin to the finest branches of the hepatic duct and lymphatics, I have termed in a former publication

"*biliary tubules*," for the reason that they carry bile, and not to have them confounded with the capillaries that carry blood. The hepatic cells are very irregular in size and form, so much so that there are hardly two exactly alike to be found; the only characteristic they all possess, in common, is their polygonal form. A nucleus, containing a nucleolus and some granules, exists in their interior; they also contain a number of granules and sometimes even fat globules. When the hepatic cells are examined while they are floating in water, their numerous surfaces can distinctly be observed, (Fig. II. *f*,) for in a quiescent state they appear almost flat. Besides the nuclei, contained within the hepatic cells, there are many that are free, (Fig. II. *g*,) which seems to indicate that the cells are formed by the primary mode of cell formation.

Lodged within the meshes of the areolar tissue of the capsules of the liver is a very extensive system of small follicular and racemose glands, the arrangement of which is peculiar. The interior of the latter is lined by an epithelium of hexagonal cells, containing a large nucleus; a number of free nuclei are interspersed. These glands join and communicate with a network of vessels, or rather ducts, forming large meshes, very irregular in size and form. (See Figs. III., IV. and V.) The diameter of these ducts varies considerably; for while in one place it may not be larger than that of a capillary blood-vessel, in another it is four or six times as large. I consider these vessels to be the ducts of the glands, as they are lined by the same epithelium of hexagonal cells. In places where the diameter of the duct is large, the epithelial cells are as large as those lining the glands; but as the former decreases, the cells also decrease in diameter. Now from this network fine branches proceed to join the larger hepatic ducts. Thus it seems that the secretion of the small glands is poured into the ducts, (forming the network of large meshes,) whence it is by their branches carried and discharged into the larger hepatic ducts, in a manner that I shall describe directly. But as the epithelium lining the ducts is the same as that of the glands, they undoubtedly elaborate the same secretion as the latter. This system of secreting glands and ducts exists especially in the capsule at the inferior surface of the liver, extending throughout the capsule of the portal vessels, and becoming more sparse as the latter become smaller in diameter; it also exists in the capsule of the hepatic veins and in the walls of the gall-bladder.

The hepatic ducts are lined by a mucous membrane with a columnar epithelium; but, as they become smaller, this epithelium is gradually changed into a squamous one. The cells composing the epithelium decrease in diameter as the ducts become smaller, until the ultimate ducts are only lined by an epithelium consisting of nuclei. When the interior of one of the larger hepatic ducts is laid open, a considerable number of small "*cul-de-sacs*" may be observed. They are formed by a duplicature or semi-circular fold of the mucous membrane. The margin of the fold is turned towards the interior of the liver, indicating that its object is to arrest the bile in the "cul-de-sacs." In the bottom of the latter are found a number of dark points, which are the orifices of the small ducts proceeding from the network of "*biliary glands*" (as they may be styled) above described. (See Fig. III. *b* and *c*.) The deduction from these facts would be, that the bile, as it flows from the interior of the liver, is arrested within the "cul-de-sacs" for the purpose of being mixed with the secretion of the system of biliary glands. Theile, a German anatomist, first described these glands, and assigned to them the office of secreting mucus. Beale considered them to be small "diverticuli" for the bile, and their office similar to that of the gall-bladder. I regarded both of these theories as erroneous; for if these were their true functions, they would be lined by a mucous epithelium. On the contrary, I am rather inclined to think that they secrete one of the constituents of the bile which becomes mixed with the latter in the "cul-de-sacs."

The finest branches of the *lymphatic vessels* of the liver originate, as has already been mentioned, within the parenchyma, in the capillary network of *biliary tubules*, being the same network in which the finest branches of the hepatic duct commence. They then join each other to form a plexus within the capsules. From this, other branches proceed, which, repeatedly joining each other, at last form the larger lymphatic vessels. They accompany the portal vein, hepatic artery and duct, to leave the organ at its inferior surface. In the common capsule, surrounding the whole organ, the finest branches of the lymphatics, after having originated from the biliary tubules, form a beautiful network of large meshes; from it larger branches proceed to form still larger ones by repeated junctures, until they at last leave the organ at its posterior surface. The finest branches of the lymphatics are as fine as, if not finer, than a capillary blood-vessel, and, like the larger ones, are provided with valves. The latter fact is proved by the numerous constrictions of their walls after they are injected with coloring matter. By means of minute injections, I have also discovered that some of the microscopic lymphatic vessels join minute hepatic ducts, and others join the network formed by the ducts of the biliary glands. The lymphatics, therefore, communicate with the hepatic ducts in three different ways. Firstly, directly by originating from the same network of *biliary tubules* in which the finest branches of the hepatic duct commence; secondly, directly by small lymphatic branches joining *small hepatic ducts;* and, thirdly, indirectly by small branches joining the network of *biliary glands,* which, in its turn, communicates with the larger hepatic ducts.

The *hepatic artery,* while accompanying the portal vein and hepatic duct, sends off small branches to supply the coats of the two latter vessels. These branches ramify within the different coats, and terminate, as anywhere else, in a capillary network, from which small veins arise, which ultimately join branches of the portal vein. In the same manner the system of biliary glands is supplied with blood. The latter organs, with their network of ducts, are surrounded by a capillary network of fine meshes, in which small branches of the artery terminate and small branches of the portal vein commence. Each glandule is embraced by a loop of capillary vessels.

The *nerves* of the liver are derived from the pneumogastric and sympathetic nerves, and form a plexus around the blood-vessels and duct. But when the branches of the latter become very small, they are only accompanied by one nervous filament. I have not traced the nerves to their termination, but I have seen their filaments or fibres still accompanying arteries as small as one-thousandth of an inch in diameter.

The *gall-bladder* is a pear-shaped sac, whose narrower extremity terminates in a duct, which joins the hepatic duct. Like the latter, the gall-bladder is lined by a mucous membrane, the inner surface of which is thrown into reticulated folds, similar in shape to the villi of the duodenum. Between the larger folds, smaller ones of the same character exist. Their object is to increase the secreting surface. The epithelium consists of columnar cells of considerably larger size than those forming that of the hepatic duct. The mucous membrane lining the duct of the gall-bladder is thrown into semicircular folds, which, alternating in position with each other, form a spiral. The object of this arrangement evidently is to arrest the too rapid flow of bile. The biliary glands, with their network of ducts, are also found around the walls of the gall-bladder and its duct. The gall-bladder has been considered to be a diverticulum for the bile; but, while acting as such, the above-mentioned folds of its mucous membrane and the extraordinary size of its epithelial cells indicate an additional function. The secretion which it furnishes may, perhaps, cause some alteration in the bile, and thus become an essential constituent of the latter.

I have thus far described the mere outlines of the microscopic anatomy of the human liver. As the object of this treatise is to present to the medical practitioner the true relationship of the component parts of the liver, as a foundation for his pathology and treatment of the diseases of that organ, I consider it unnecessary to describe and discuss the numerous minutiæ which are interesting only to the anatomist, for it would complicate the subject. In the memoir, spoken of in the "prefatory remarks," they are fully described and illustrated.

*Remarks on the Physiology and Pathology of the Liver.*

In connection with the anatomy of the liver, I now propose to survey, briefly, its physiology and pathology. At present, it is universally understood and accepted that the liver secretes bile and forms sugar. But the exact manner in which this is effected is still only obscurely known. A portion of the blood, carried to this gland by the hepatic artery, is expended in supplying the coats of the blood-vessels, ducts and lymphatics, and the nerves and biliary glands with nutrient materials, and, after having done so, returns as venous blood to branches of the portal vein. But the rest, if not the greater portion, is discharged by the terminal branches of the artery into the same capillary blood-vessels into which the terminal branches of the portal vein discharge their venous blood; and it is thus that the arterial and venous blood become mixed. The circulation of the liver is, therefore, somewhat similar to that of the fœtus and reptiles. If we now consider the greater rapidity with which the blood moves in the artery than in the vein, we might infer that the quantity of arterial blood carried to the capillaries is, at least, equal to that carried there by the portal vein. Thus, as far as the arterial blood is concerned, the liver corresponds to all other secreting glands, *i. e.*, in being supplied by arterial blood. But in as far as the venous blood is concerned, it deviates from all the rest. Now, the question arises: does the arterial blood contribute its elements to the formation of bile? Ever since I commenced to investigate the subject, I have been inclined to think that it does. And especially lately, my view has become more confirmed by a suggestion made to me by my friend, Surgeon J. F. Fauntleroy, a member of one of the Army Medical Examining Boards. His idea is, that, after the principles essential to the elaboration of bile have been abstracted from the blood, carbon, oxygen and hydrogen, in quantity corresponding to the equivalents of sugar, would be set free, and, being in a nascent state, readily combine to form sugar, within the blood-vessels, which, as soon as formed, was carried immediately to the heart. Now, it is a difficult matter to determine where the sugar is formed—whether by mere chemical laws in the blood-vessels, as Dr. Fauntleroy suggests, or by a higher vital process, that of organic cells. The opinion I hold is in favor of the former. For if the sugar was formed within the cells, it would have to be discharged by them, and again penetrate the walls of the capillaries to reach the blood, which would be a complicated process. However, in regard to this, it might be answered that the presence of sugar in the parenchyma of the liver is proved by chemical test. Undoubtedly it is. But does the parenchyma of the liver consist solely of secreting cells? Are there no blood-vessels present? A glance at the diagrams will show how intimately they are interwoven with each other. And where is the anatomist who could dissect them so nicely as not to remain connected with each other, and in a quantity large enough to be subjected to chemical test? If I remember rightly, one of the experiments of that distinguished physiologist, Claude Bernard, the very man who first discovered the presence of sugar in the liver, was that, in order to completely remove the blood from the organ, he inserted canulæ into the portal vein and hepatic artery, connected with a long elastic tube. The latter was then elevated to a certain height, where it communicated with a reservoir of water. Thus, the water would descend through the tube into the blood-vessels by its own weight, which, of course, could be regulated by appropriate stop-cocks, so as to keep up a constant current through the organ. After this had been continued for several days, the experimentor, thinking now that all the blood must have been removed, took some of the parenchyma to subject it to chemical test, by which, as might be expected, he detected still the presence of sugar. This sugar, however, was undoubtedly contained in the blood remaining behind in the blood-vessels. For there exists no means by which the blood may be completely removed from an organ except by chemical injections, which, of course, will also in some degree destroy the latter. The blood that remains in the smaller blood-vessels after death, imperceptible in quantity as it may be, is yet sufficient to present one of the greatest obstacles in the process of minute injection. Every anatomist, who is experienced and skilled in this department, will agree with me on that point. In my own experience, which has been extensive, I have at least found it so. I will for the present, until the contrary is proved, admit then that the sugar is formed within the blood, and is consequently no secretion, but a mere formation. But I am also aware that there are many facts existing, which still might, in some degree, render this admission untenable. Some of them are that sugar, (in the normal condition of the animal,) is found only in the blood, while it is circulating through the liver, and for a certain distance beyond. And that the peculiar kind of food, as Bernard clearly proved, whether nitrogenous, starchy, fatty, &c., on which the animal is fed, does not affect nor arrest the formation of sugar. But even admitting and considering these facts, they would in no way materially affect Dr. Fauntleroy's suggestion; for the food of which it is composed has first to undergo a considerable alteration during the process of digestion, before it can be absorbed by the capillaries (and *perhaps* the venules) of the abdominal viscera to be carried to the liver, and when reaching the latter, it has been completely reduced to the simplest organic elements, which are common to all varieties of food. It would be immaterial, therefore, whether these principles, as the albuminous, for example, were at one time constituents of meat or of vegetables. In every case, after the principles essential to the formation of bile were abstracted by organic cell action, the elements remaining behind in a nascent state might continue to form sugar. I am inclined to think that both venous and arterial blood furnish the constituents of the bile. Still, if this could be denied, I would rather decide in favor of the arterial, because the bile is more a secretion than an excretion, and for the reason that all other secreting glands are supplied only by arterial blood. It is accepted by physiologists that the liquid portion of the bile is subservient to the process of digestion, and that it is ultimately re-absorbed in the intestines, the coloring matter only being excrementitious, and carried off with the fæces. It might be suggested, therefore, that both the arterial and venous blood contribute elements to the secretion of bile, while the venous alone, containing a large quantity of carbon, contributes to the formation of sugar, and also furnishes the coloring matter, the latter being most likely derived from red blood-corpuscles, disintegrated during their passage through the spleen. But there is still another office the arterial blood evidently has to perform: this is to promote the organic cell action; or, in other words, to form the cell and keep it alive by furnishing oxygen, and also the material from which the cell walls are built. To the extent of my knowledge, there is no instance in which cells or tissues are formed from a fluid exuding from the veins. The exact process by which the secretion of bile is carried on is difficult to determine, and I by no means pretend that the explanation I shall give is strictly correct, but from the facts I have observed, the following seems to me to be the most probable: In referring to what I have said on the subject of secretion, we find that the liquor sanguinis, exuding from the capillary blood-vessels, is the plasma from which all cells and their secretions are formed, and it contains the elements essential to that purpose. I also stated that, as soon as the cell is fully formed, the chemical process of secretion, under the organic influence of the nucleus, commences within its cavity, and that the cell bursts to discharge its contents as soon as the secretion is finished. The same I consider to take place in the liver. A portion of the liquor sanguinis, containing the elements essential to the secretion of bile, and the formation of cell-walls, exudes from the capillary blood-vessels. From this the cells are formed, and within their cavity the bile is elaborated. When the latter is finished, the cells burst, and their contents are discharged into the interspaces of the capillaries. The bile is now absorbed by the network of "biliary tubules," and carried to the finest hepatic ducts, from which it finally passes into the larger ones to find its outlet from the liver. (See Figure II.) The debris, or remnants of the cell-walls, is dissolved in the secretion, but the nuclei remain, probably to serve for the formation of new cells, by having another cell-wall formed around them. This latter view I have taken by finding many free nuclei in examining portions of the parenchyma. It is true I also observed cells and their nuclei in a state of partial subdivision, which would indicate a multiplication of division; but these instances are so few that I regard them as exceptions to the rule. Now in all tissues or secreting organs, in a normal condition of things, all the plasma that exosmoses from the blood-vessels is not consumed either for the purpose of secretion or reconstruction, but some remains behind as a surplus. What then becomes of it? It is re-absorbed by the lymphatics along with the waste that at the same time is taking place.

In order to understand this physiological process properly, we must not imagine it commences at one time and terminates at another, when, after the bursting of all the cells, and the removal of the bile by the hepatic ducts, the liver would necessarily collapse, and its size diminish. On the contrary, the process is going on continually, to a certain extent, but is especially active during digestion. And to corroborate this fact, I have only to refer to the various sizes, or stages of development, in which the hepatic cells are found when a small portion of the parenchyma of the liver is examined under the microscope. (See Figure II. *f*)

There is another important fact yet, in connection with the physiology of the liver, brought to light by my researches. It is the communication existing between the lymphatics and the hepatic ducts. The question, what is the object of this triple communication? arises, but up to this day I have not been able to solve it. However, my venerable friend, Prof. Samuel Jackson, of the University of Pennsylvania, shortly after I made the discovery, built an hypothesis upon it. He presumes that the nature of the bile, after having been secreted by the cells, is very viscid, and that in order to render it more fluid, and promote its flow through the hepatic ducts, the lymphatics pour some of their watery contents into the latter. That the bile, while contained within the hepatic cells, is of a very viscid nature, I have proved satisfactorily by the tearing of hepatic cells by means of my microscopic dissector (a delicate instrument constructed for similar purposes), under high magnifying powers of the compound microscope. In such instances, when the cell-wall was torn and drawn asunder, the contents could be drawn into filaments, like thick molasses.

The probable function of that system of "biliary glands" I have already stated in connection with their anatomy. I will, however, repeat here that I regard them as secreting some of the fluid constituents of the bile, which are discharged and mixed with the latter in those small "cul de sacs" already described.

[To be continued in next number.]

## Figures and Tables

**Fig. I. f1:**
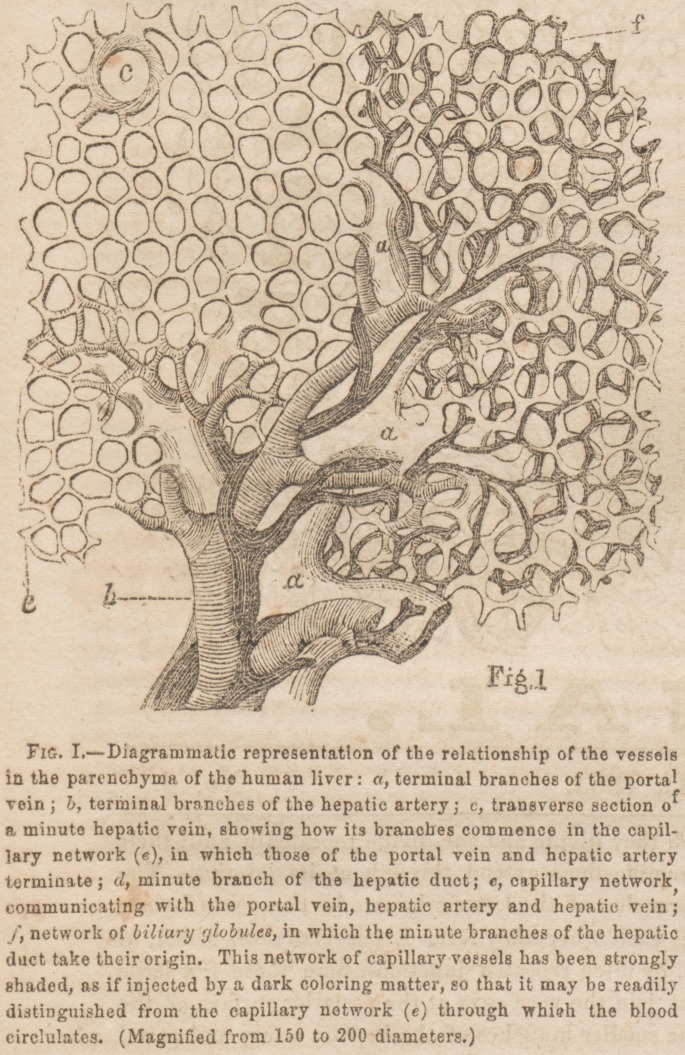


**Fig. II. f2:**
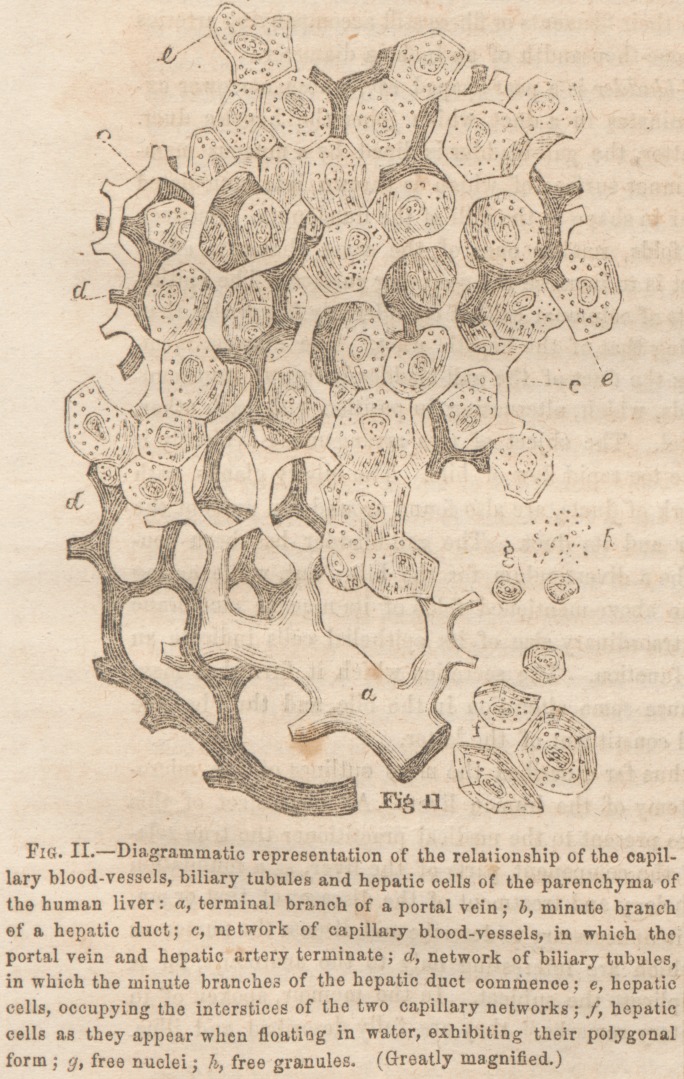


**Figure f3:**